# Longitudinal changes in wellbeing amongst breastfeeding women in Australia and New Zealand during the COVID-19 pandemic

**DOI:** 10.1007/s00431-022-04580-y

**Published:** 2022-08-17

**Authors:** Vanessa S. Sakalidis, Alethea Rea, Sharon L. Perrella, Jacki McEachran, Grace Collis, Jennifer Miraudo, Stuart A. Prosser, Lisa Y. Gibson, Desiree Silva, Donna T. Geddes

**Affiliations:** 1 School of Molecular Sciences, University of Western Australia, Perth, WA 6009 Australia; 2 Mathematics and Statistics, Murdoch University, Perth, WA Australia; 3 One For Women, Perth, WA Australia; 4 Telethon Kids Institute, Perth, WA Australia; 5 The University of Western Australia, Perth, WA Australia; 6 School of Health and Medical Sciences, Edith Cowan University, Perth, WA Australia; 7 Health and Medical Sciences, The University of Western Australia, Perth, WA Australia; 8 Joondalup Health Campus, Perth, WA Australia

**Keywords:** Breastfeeding, COVID-19, SARS-CoV-2, Mental health, Depression, Anxiety

## Abstract

The COVID-19 pandemic has impacted new mothers’ wellbeing and breastfeeding experience. Women have experienced changes in birth and postnatal care and restricted access to their support network. It is unclear how these impacts may have changed over time with shifting rates of infection and policies restricting movement and access to services in Australia and New Zealand. This study investigated the longitudinal effect of the COVID-19 pandemic on breastfeeding and maternal wellbeing in Australia and New Zealand. Mothers (*n* = 246) completed an online survey every 4 weeks for 6 months that examined feeding methods, maternal mental wellbeing, worries, challenges, and positive experiences during the pandemic. Mothers maintained high full breastfeeding rates at 4 months (81%) which decreased to 37% at 6 months. Perceived low milk supply contributed to the earlier cessation of full breastfeeding. Poor infant sleep was associated with stress, perinatal anxiety, mental wellbeing, and breastfeeding status. Although mothers initially reported that lockdowns helped with family bonding and less pressure, prolonged lockdowns appeared to have adverse effects on access to social networks and extended family support.

*   Conclusion*: The results highlight the changing dynamic of the pandemic and the need for adaptable perinatal services which allow mothers access to in-person services and their support network even in lockdowns. Similarly, access to continuous education and clinical care remains critical for women experiencing concerns about their milk supply, infant sleep, and their own wellbeing.

**Table:** 

**What is Known:** *• The COVID-19 pandemic and lockdown restrictions have significantly affected perinatal mental health, disrupted maternal services, and subsequent breastfeeding.*	
**What is New:** *• In Australia and New Zealand, breastfeeding women experienced challenges to their mental wellbeing, sleep, and breastfeeding, which was likely exacerbated over time by the pandemic. Lockdowns, while initially beneficial for some families, became detrimental to maternal support and wellbeing.*

**Supplementary Information:**

The online version contains supplementary material available at 10.1007/s00431-022-04580-y

## Introduction

The initiation and continuation of breastfeeding remain critical to infants’ health and development during the COVID-19 pandemic [1]. Evidence has demonstrated that antibodies isolated in the breastmilk of infected mothers [2–4] and mothers after vaccination [5–8] have robust secretory IgA activity specific to SARS-CoV-2, potentially providing infants with additional protection against the virus. Unfortunately, the pandemic has negatively impacted mothers’ wellbeing and breastfeeding experience globally despite this evidence [9].

Since the pandemic began, social distancing and stay-at-home measures have intensified the stress experienced by women perinatally. Mothers have experienced a disproportionate burden of household tasks, childcare responsibilities, and economic insecurity [10, 11]. Moreover, the pandemic has restricted access and delivery of perinatal services, face-to-face postnatal care, and social support leading to isolation and loneliness [11–13]. Consequently, unprecedented increases in perinatal anxiety and depression rates have occurred [11, 14–18].

Early in the pandemic, our cross-sectional study confirmed breastfeeding women experienced challenges to their mental wellbeing in Australia and New Zealand (NZ) [19]. Women affected by the pandemic for longer durations of their pregnancy and those living in regions with higher COVID-19 infection rates experienced poorer mental health. Internationally, for some women, the pandemic and lockdowns resulted in less pressure and more family support to continue breastfeeding [16–18]; however, others highlighted reduced access to support directly contributed to early weaning [17].

While data show that the pandemic has impacted breastfeeding women, this may have changed over time with shifting infection rates and policies restricting movement and access to services in Australia and NZ. Since March 2020, regions of Australia and NZ have endured international travel bans, state border closures, and multiple long-term lockdowns [20, 21]. It is unclear how women have adapted to their ‘new normal’ over time or if breastfeeding challenges have changed during the pandemic.

This study aimed to investigate the longitudinal effect of the pandemic on breastfeeding and maternal wellbeing in Australia and NZ. Specifically, we aimed to understand how COVID-19 restrictions have impacted stress, anxiety, mental health, and breastfeeding patterns over time. We also examined the longitudinal impact on wellbeing through qualitative reports of worries and challenges.

## Materials and methods

### Participants

We conducted an online longitudinal survey of breastfeeding women between June 2020 and May 2021. Eligible participants lived in Australia or NZ and were fully (receiving only breastmilk) or partially breastfeeding a healthy infant aged 0–7 months. Women were excluded if their infant was born < 37-week gestation or had a health condition that affected breastfeeding. Participants provided online informed consent for the study, approved by The University of Western Australia Ethics Committee (RA4206286 and RA4204023).

### Procedure

Participants completed an identical survey monthly (six times) over a period of 6 months. The survey contained closed questions detailing maternal and infant demographic and health information, breastfeeding history, COVID-19 behaviour, and open-ended questions about the mother’s experiences during the pandemic. Several scales assessed breastfeeding status, maternal wellbeing, family support, and financial hardship.

### Demographic, health information, and breastfeeding history

Participants reported maternal age, education, ethnicity, parity, marital status, birth details, infant age, and maternal and infant health status. Breastfeeding history included previous breastfeeding experiences and current breastfeeding problems.

### COVID-19 behavioural aspects

Behavioural aspects associated with work and home life during the pandemic were recorded. Questions detailed maternal employment status; whether they were a healthcare worker, if they worked from home or outside of the house, exercised outside and left their home in the last 7 days or avoided face-to-face contact with friends or family over 65 years, and how frequently they checked COVID-19-related news, and whether they were self-isolating.

### Maternal and infant wellbeing scales

#### Infant Feeding Practices Study Questionnaire (IFPS II)

An adapted version was used to determine breastfeeding experiences, including breastfeeding duration, formula use, and the timing and reasons for stopping breastfeeding [22, 23]. Using a 4-point scale, mothers rated the importance of certain factors which influenced their decision to cease breastfeeding.

#### Perceived Stress Scale (PSS)

It is a validated [24] 10-item scale that assessed how unpredictable, uncontrollable, and overloaded participants had found their lives over the last month. Participants rated four positively worded items and six negatively worded items using a Likert scale, with higher scores indicating higher levels of perceived stress.

#### General Functioning subscale (GF6 +) of the McMaster Family Assessment Device (FAD)

It is a validated 6-item subscale of the FAD scale that characterises family functioning [25]. The GF6 + uses a 4-point scale with higher scores indicating worse family functioning [26].

#### Hardship scale

Financial stress was assessed using a 6-item scale previously utilised in Australia [27]. A ‘yes’ response to any of the questions was categorised as experiencing hardship.

#### Mental Health Continuum-Short Form (MHC-SF)

It consists of 14 items which consist of three subscales assessing the social, psychological, and emotional levels of mental wellbeing. Items are rated using a 6-point response scale ranging from 1 (never) to 6 (every day) to indicate the frequency of experiencing various measures of wellbeing over the previous month. From the subscale scores, a total is calculated, with higher scores indicating greater levels of wellbeing. Total scores are then catergorised as either flourishing, moderate, or languishing mental wellbeing [28].

#### Perinatal Anxiety Screening Scale (PASS)

This validated 31-item scale assesses perinatal anxiety using four subscales that measure general worry and specific fears; perfectionism, control, and trauma; social anxiety, acute anxiety and adjustment over the past month. Based on a 4-point scale, higher scores indicate higher levels of perinatal anxiety [29, 30].

#### Brief Infant Sleep Questionnaire (BISQ)

This validated scale uses seven items to evaluate infant sleep patterns and parents’ perceptions of their infant’s sleep [31]. Items assess nighttime and daytime sleep duration, night waking frequency, wakefulness duration, sleep-onset time, settling time and method, and whether the parent considers their infant’s sleep as not a problem, a small, or very serious problem.

### Worries and concerns open-text questions

Participants completed open-text questions describing their worries, concerns, and any positive experiences resulting from the pandemic [32]. Participants were asked: ‘What are your three biggest worries right now?’; ‘Can you tell us about a challenge you have faced in the last two weeks?’, and ‘Can you tell us how lockdown has made any parts of your life easier or more enjoyable?’.

### Statistical analysis

Cox proportional hazard modelling was used to investigate associations with the time to not fully breastfeeding (partial or stopping). Associations were considered for: overtime during the pandemic (survey one to six), maternal factors (age, self-reported anxiety, and depression, parity, number of days pregnant since 1 March 2020), infant factors (age, in childcare), breastfeeding problems (blocked ducts, sore nipples, attachment difficulties, nipple damage, mastitis, an oversupply of milk, low milk supply, nipple shield use), employment history (impacted by COVID-19, healthcare worker, employed but on maternity leave, working outside the home), feeding (introduction of complementary foods, introduction of infant formula, current intended breastfeeding duration), sleep (if infant sleep is a perceived problem, infant’s sleep duration in the day/night, and average night waking frequency), financial hardship, exercising out of the home, family functioning (GF6 + FAD), and visiting of those > 65 years of age during the lockdown. Also, maternal wellbeing was assessed as an explanatory variable (PSS, PASS, MHC-SF total score, and categorical), and the mother’s comments on worries, challenges, and impact of lockdown were based on the qualitative coding below.

Generalised linear mixed models were used to assess the factors influencing breastfeeding and maternal wellbeing. We considered five response variables: breastfeeding status (full breastfeeding yes/no), and the total and categorical scores of PSS (high/moderate vs. low-stress scores), PASS (high/moderate vs. low), and MHC-SF (flourishing, moderate, or languishing mental health). For each response, univariate models with explanatory variables as described above were considered. A random effect for mother was included in all models.

For each univariate model, variables with a *p*-value < 0.1 were retained for multivariate modelling. Missing data were accounted for with missing case analysis, and the significance level was set at 0.05. Model output (coefficient or OR, CI, and *p*-value) was reported for multivariable models. All quantitative data were analysed using R (R Development Core Team, 2017).

Qualitative responses were analysed thematically. Responses were coded based on theme development from the responses’ content and were further divided into subthemes. Percentages were reported for each theme found within the responses concerning worry, challenges, and lockdown benefits.

## Results

### Participant characteristics and demographics

**Fig. 1:**
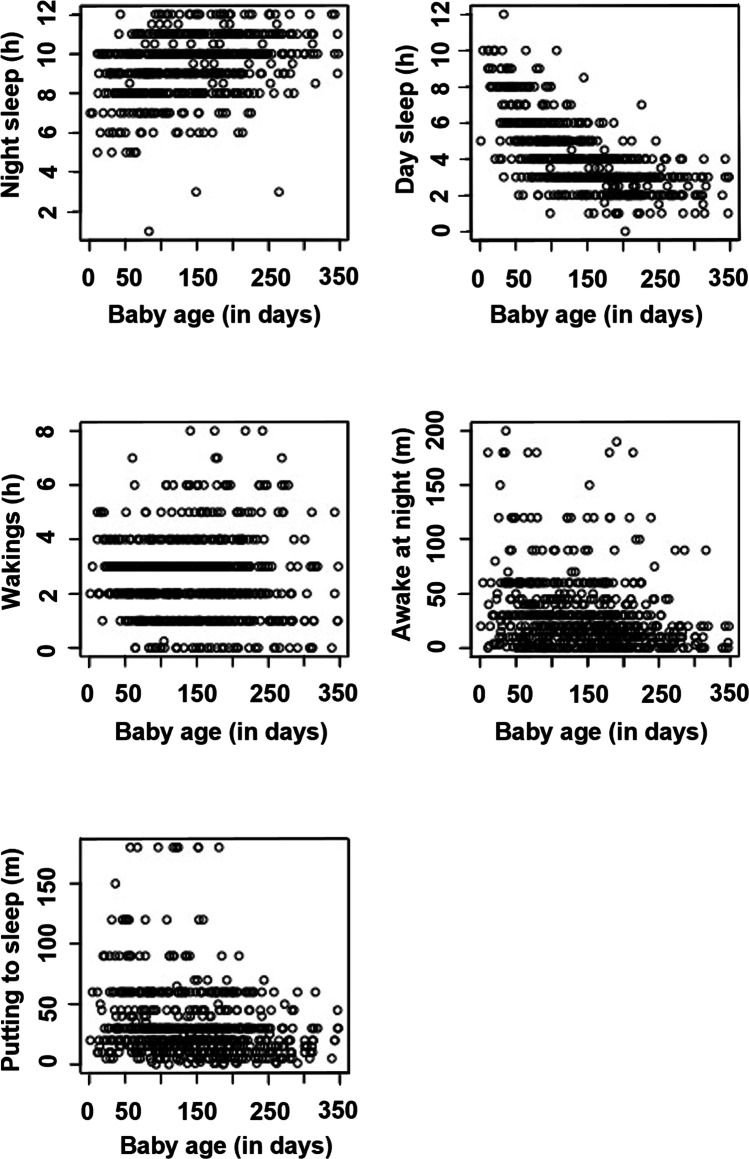
Sleep characteristics by infant age (days)

Of the 246 participants in the first survey (Table 1), most were university-level educated (76%), healthcare professionals (62%), and were employed but on maternity leave (80%), infants were 91 ± 57 days old, and 82% were fully breastfeeding (Table 2). Considering breastfeeding by infant age, across surveys, 93% were fully breastfeeding at 1 month (*n* = 68), 87% at 3 (*n* = 95), 81% at 4 (*n* = 99), 62% at 5 (*n* = 95), and 37% at 6 months respectively. Women most frequently reported sore nipples during breastfeeding (33%) and anxiety (25.6%) as health issues. Around one-third of women perceived their infant’s sleep as a problem, and infants woke 2.5 times and slept 9–10 h at night consistently across surveys (Fig. 1).

**Table 1:** Demographics and participant characteristics

Variable	Mean ± SD, Missing or Count (%)
*Infant characteristics*	
Infant age (days)	91.2 (57.6), 0
Birth gestation (weeks)	39.4 (1.1), 0
Birth weight (g)	3456.6 (427.5), 1
Birth length (cm)	50.8 (2.4), 15
*Maternal characteristics*	
Maternal age (years)	32.8 (4.2), 0
Parity	
Primiparous	116 (47.2)
Multiparous	130 (52.8)
Previously breastfed duration	17.4 (12.8), 116
Marital status	
Married or de facto	240 (97.6)
Never married or de facto	5 (2)
Separated or divorced	1 (0.4)
Region	
Western Australia	126 (51.2)
Victoria	20 (8.1)
New South Wales	24 (9.8)
Rest of Australia	16 (6.5)
New Zealand	60 (24.4)
Education	
Bachelor degree or above	187 (76)
Certificate level IV	13 (5.3
Certificate level I–III	9 (3.7)
Diploma	19 (7.7)
High school	18 (7.3)
Ethnicity	
Aboriginal or Torres Strait Islander	3 (1.1)
Australian	183 (68.3)
British	30 (11.2)
Asian	8 (3.0)
European	20 (7.5)
Other	24 (9.0)
Maternal health issues	
Anxiety	63 (25.6)
Depression	26 (10.6)
Diabetes (diagnosed before this pregnancy)	5 (2)
Fertility issues requiring assisted reproduction for this pregnancy	18 (7.3)
Thyroid disorder	9 (3.7)
Insulin resistance	4 (1.6)
Polycystic ovarian syndrome	18 (7.3)
No health conditions	127 (51.6)
Other	31 (12.6)
Breastfeeding problems	
Sore nipples	83 (33.7)
Nipple damage	47 (19.1)
Attachment difficulties	49 (19.9)
Nipple shield use	43 (17.5)
Blocked ducts	27 (11)
Mastitis	26 (10.6)
Low milk supply	23 (9.3)
Oversupply	31 (12.6)
Planned breastfeeding duration (months)	15.8 (8.4)

**Table 2:** Breastfeeding, maternal health issues, wellbeing scales, and COVID-19 behaviour across surveys (Count (%) or Mean ± SD, Missing)

Variables	Survey 1	Survey 2	Survey 3	Survey 4	Survey 5	Survey 6
Breastfeeding status						
Fully breastfeeding	202 (82.1)	111 (73.5)	75 (65.8)	45 (51.1)	24 (41.4)	12 (23.1)
Partial breastfeeding	44 (17.9)	30 (19.9)	31 (27.2)	35 (39.8)	31 (53.4)	36 (69.2)
Missing	NA (NA)	10 (6.6)	8 (7)	8 (9.1)	3 (5.2)	4 (7.7)
Introduced infant formula						
No	24 (9.8)	21 (13.9)	22 (19.3)	28 (31.8)	25 (43.1)	31 (59.6)
Yes	20 (8.1)	11 (7.3)	12 (10.5)	10 (11.4)	8 (13.8)	8 (15.4)
Not applicable — fully breastfeeding	202 (82.1)	119 (78.8)	80 (70.2)	50 (56.8)	25 (43.1)	13 (25)
Introduced complementary food						
No	20 (8.1)	8 (5.3)	6 (5.3)	1 (1.1)	2 (3.4)	NA (NA)
Yes	24 (9.8)	24 (15.9)	28 (24.6)	37 (42)	31 (53.4)	39 (75)
Not applicable — fully breastfeeding	202 (82.1)	119 (78.8)	80 (70.2)	50 (56.8)	25 (43.1)	13 (25)
Hardship						
No	198 (80.5)	122 (80.8)	92 (80.7)	70 (79.5)	48 (82.8)	44 (84.6)
Yes	48 (19.5)	22 (14.6)	17 (14.9)	13 (14.8)	9 (15.5)	7 (13.5)
Missing	NA (NA)	7 (4.6)	5 (4.4)	5 (5.7)	1 (1.7)	1 (1.9)
GF6 + FAD score	9.6 (3.4), 0	9.8 (3.4), 4	10.1 (3.8), 5	10 (3.5), 5	9.5 (3.2), 1	9.7 (3.7), 0
MHC score						
Emotional (score: /15)	12.6 (2.4), 0	12.2 (2.5), 7	12 (2.5), 5	11.7 (2.8), 5	12.1 (2.8), 1	12.3 (2.8), 1
Social (score: /25)	13.4 (5.2), 0	13.2 (5.3), 7	13.7 (5), 5	13.9 (5), 5	14.5 (5.9), 1	15.4 (5.8), 1
Psychological (score: /30)	22.6 (5.1), 0	21.7 (5.3), 7	21.5 (5.2), 5	21.9 (5.3), 5	21.9 (5.9), 1	22.1 (5), 1
MHC categories						
Flourishing	111 (45.1)	54 (35.8)	48 (42.1)	32 (36.4)	26 (44.8)	24 (46.2)
Languishing	4 (1.6)	3 (2)	1 (0.9)	NA (NA)	2 (3.4)	NA (NA)
Moderately mentally healthy	131 (53.3)	87 (57.6)	60 (52.6)	51 (58)	29 (50)	27 (51.9)
Missing	NA (NA)	7 (4.6)	5 (4.4)	5 (5.7)	1 (1.7)	1 (1.9)
PSS score						
Average	16 (6.4), 0	15.6 (6.9), 4	15.8 (7.1), 5	15.7 (7.6), 4	15.8 (7.8), 0	14.8 (8), 0
Low (score: 0–13)	11 (4.5)	9 (6)	9 (7.9)	8 (9.1)	6 (10.3)	6 (11.5)
Medium (score: 14–26)	93 (37.8)	54 (35.8)	40 (35.1)	33 (37.5)	28 (48.3)	26 (50)
High (score: 27–40)	142 (57.7)	84 (55.6)	60 (52.6)	43 (48.9)	24 (41.4)	20 (38.5)
PASS score						
Average	21.9 (14.7), 12	21.1 (14.7), 10	21.4 (15.9), 6	21.3 (14.8), 5	21.9 (14.7), 12	21.1 (14.7), 10
Minimal anxiety symptoms (score: 0–20)	81 (32.9)	43 (28.5)	28 (24.6)	21 (23.9)	19 (32.8)	10 (19.2)
Mild‚ moderate anxiety symptoms (score: 21–41)	130 (52.8)	85 (56.3)	66 (57.9)	52 (59.1)	32 (55.2)	36 (69.2)
Severe anxiety symptoms (score: 42–93)	23 (9.3)	13 (8.6)	14 (12.3)	10 (11.4)	6 (10.3)	5 (9.6)
Missing	12 (4.9)	10 (6.6)	6 (5.3)	5 (5.7)	1 (1.7)	1 (1.9)
Employment impacted by COVID						
No	232 (94.7)	143 (95.3)	112 (98.2)	82 (94.3)	57 (98.3)	48 (92.3)
Yes	13 (4.6)	7 (1.7)	2 (1.8)	5 (5.7)	1 (1.7)	4 (7.7)
Missing	1 (0.4)	1 (0.7)	NA (NA)	1 (1.1)	NA (NA)	NA (NA)
Work as a healthcare professional						
No	91 (37.0)	61 (40.4)	44 (39.2)	34 (38.6)	21 (36.2)	22 (42.3)
Yes	153 (62.2)	90 (59.6)	68 (59.6)	54 (61.4)	36 (62.1)	30 (57.7)
Missing	2 (0.8)	NA (NA)	2 (1.2)	NA (NA)	1 (1.7)	NA (NA)
Employed and on maternity leave						
No	50 (20.3)	36 (23.8)	31 (27.2)	28 (31,8)	22 (37.9)	21 (40.4)
Yes	195 (79,3)	114 (75,5)	83 (72,8)	59 (67)	36 (62,1)	31 (59.6)
Missing	1 (0.4)	1 (0.7)	NA (NA)	1 (1.1)	NA (NA)	NA (NA)
Exercise outside of home in the last 7 days						
No	39 (15.9)	18 (11.9)	14 (12.3)	15 (17)	11 (19)	9 (17.3)
Yes	207 (88.1)	132 (87.4)	100 (87.7)	73 (83)	47 (81)	43 (82.7)
Missing	NA (NA)	1 (0.6)	NA (NA)	NA (NA)	NA (NA)	NA (NA)
Avoid contact with someone over 65 years						
No	149 (60.6)	94 (62.3)	77 (67.5)	65 (73.9)	48 (82.8)	47 (90.4)
Yes	64 (26)	40 (26.5)	25 (21.9)	14 (15.9)	4 (6.9)	2 (3.8)
Not applicable	33 (13.4)	17 (11.3)	12 (10.5)	9 (10.2)	6 (10.3)	3 (5.8)
Infant in childcare						
No	241 (98)	148 (98)	111 (97.4)	85 (96.6)	55 (94.8)	50 (96.2)
Yes	5 (2)	3 (2)	3 (2.6)	3 (3.4)	3 (5.2)	2 (3.8)

### Time to stopping full breastfeeding

Shorter full breastfeeding duration was associated with low milk supply (*p* < 0.001), increased infant day sleep duration (*p* < 0.005), primiparity (*p* < 0.001), and being pregnant more days during the pandemic (*p *< 0.001). Overtime (in later surveys), women were also more likely to have ceased full breastfeeding (*p* < 0.005) (Table 3).

**Table 3:** Multivariate models for Time to partial breastfeeding, PASS score and category, PSS score and category, MHC score and category

Response		Multivariate Modelling*		
	Variable	Coeff/OR	CI	P-value
Time from fully to partial BF	Survey number	1.35	(0.1. 1.67)	0.004
	Parity	0.55	(0.36, 0.86)	0
	BF problems (low milk supply)	3.66	(1.86, 7.20)	0
	Day sleep duration	1.29	(1.09, 1.53)	0.003
	Days pregnant during the pandemic	0.993	(0.986, 1.00)	0
PSS	Intercept	18.32	(15.66, 20.98)	0
	Breastfeeding problems- oversupply	1.76	(0.19, 3.33)	0.026
	Sleep not a problem at all problem	3.44	(1.78, 5.1)	0.86
	Sleep a serious problem	0.06	(-0.64, 0.77)	0
	Worry (parenting and relationships)	-0.75	(-1.41, -0.1)	0.02
	Lockdown benefits (no benefit)	1.5	(0.12, 2.88)	0.03
	MHC Score	-0.15	(-0.2, -0.11)	0
	PASS score	0.23	(0.2, 0.27)	0
PSS Categories	Intercept	2.77	(0.36, 21.38)	0.32
	Maternal Health (Depression)	10.06	(1.68, 60.28)	0.009
	Parity	2.61	(1.19, 5.72)	0.014
	Over 65 other	2.51	(0.83, 7.6)	0.096
	Over 65 Yes	2.33	(1.05, 5.15)	0.03
	FAD category	3.05	(1.05, 8.84)	0.036
	Night sleep duration	0.72	(0.59, 0.89)	0.002
	Challenges (financial)	0.13	(0.04, 0.47)	0.0015
	MHC category	5.81	(2.87, 11.75)	0
	PASS category	14.26	(6.73, 30.2)	0
MHC	Intercept	59.2	(57.03, 61.37)	0
	Healthcare worker	2.3	(0.25, 4.36)	0.026
	FAD category	-2.79	(-4.45, -1.13)	0.001
	PSS score	-0.43	(-0.56, -0.31)	0
	PASS score	-0.21	(-0.27, -0.14)	0
MHC Categories	Intercept	1.07	(0.38, 2.98)	0.9
	Healthcare worker	0.24	(0.08, 0.71)	0.009
	Sleep not a problem at all problem	2.41	(0.31, 18.84)	0.014
	Sleep a serious problem	0.4	(0.19, 0.84)	0.392
	PSS category	7.75	(3.21, 18.72)	0
	PASS category	2.74	(1.18, 6.35)	0.017
PASS Score	Intercept	42.08	(29.88, 54.28)	0
	Maternal age	12.65	(6.1, 19.19)	0
	Maternal health (anxiety)	0.78	(0.65, 0.91)	0
	Education Diploma	2.77	(-1.79, 7.33)	0.226
	Certificate level IV	-0.28	(-6.03, 5.47)	0.923
	Certificate level I-III	2.35	(0.54, 4.16)	0.01
	High School	-1.1	(-6.52, 4.32)	0.686
	Sleep a serious problem	-1.26	(-4.19, 1.67)	0.392
	Sleep not a problem at all problem	-2.01	(-3.27, -0.75)	0.0015
	FAD category	2.35	(0.54, 4.16)	0.0096
	MHC Score	-0.26	(-0.34, -0.17)	0
	PSS score	0.78	(0.65, 0.91)	0
PASS Categories	Intercept	4.63	(0.1, 218.02)	0.426
	Maternal health (Anxiety)	5.61	(1.87, 16.83)	0.0017
	Education Certificate level I-III	21.64	(1.68, 278.2)	0.016
	Certificate level IV	0.16	(0.02, 1.36)	0.087
	Diploma	12.53	(2.47, 63.61)	0.002
	High School	0.44	(0.07, 2.87)	0.383
	FAD category	3.44	(1.33, 8.86)	0.009
	Time to put baby to sleep	1.02	(1, 1.03)	0.007
	Worry (household day to day)	2.14	(1.07, 4.31)	0.029
	Challenges (financial)	4.83	(1.25, 18.66)	0.019
	Challenges (baby/child health)	2.61	(1.13, 6.05)	0.022
	PSS categories	15.16	(6.81, 33.76)	0
	MHC categories	2.67	(1.29, 5.5)	0.007

^*^Variables *p* < 0.1 in univariate models were retained for multivariate modeling (Univariate modeling available in supplementary material)

### Perceived stress score

Higher PSS scores were associated with oversupply (*p* = 0.026), perception of infant sleep being a serious problem (*p* < 0.001), higher PASS score (*p* < 0.001), and stating no benefit of lockdown (*p* = 0.03). Lower PSS scores related to higher mental wellbeing scores (*p* < 0.001) and reports of worry about parenting/family relationships (*p* = 0.02).

When PSS was considered as a categorical variable, high/moderate stress was associated with maternal depression (*p* = 0.009), multiparity (*p* = 0.014), visiting over 65 s during COVID-19 (*p* = 0.03), poorer family function (*p* = 0.036), being languishing or moderately mentally healthy (*p* < 0.001), and mild/moderate perinatal anxiety scores (*p* < 0.001). High/moderate PSS was less common amongst women who reported longer infant night sleep duration (*p* = 0.002) and those reporting financial challenges (*p* = 0.0015).

### Mental wellbeing

Poorer mental wellbeing assessed by the MHC-SF was related to poorer family functioning (*p* < 0.001) and higher PSS and PASS scores (*p* < 0.001). Higher mental wellbeing was associated with working in healthcare (*p* = 0.026). When considering mental wellbeing as a categorical variable, being languishing or moderately mentally healthy increased for women reporting high/moderate stress (*p* < 0.001), and moderate/severe perinatal anxiety (*p* = 0.017). The odds were reduced amongst those working in healthcare (*p* = 0.009) and those reporting infant sleep as ‘not a problem’ (*p* = 0.014).

### Perinatal Anxiety Screening Score

Higher PASS scores were related to maternal anxiety, higher stress scores, lower mental health scores (*p* < 0.001), poorer family functioning (*p* = 0.0096) and, education level (certificate I–III, *p* < 0.01). Lower PASS scores were associated with older mothers (*p* < 0.001) and those reporting infant sleep as not a serious problem (*p* = 0.0015).

When considered as a categorical variable, severe/moderate perinatal anxiety was associated with self-reported anxiety (*p* = 0.017), poorer family functioning (*p* = 0.009), education levels (certificate I–III, *p* = 0.01; diploma, *p* = 0.002), and longer durations to settle the infant to sleep (*p* = 0.007). Worry about the household (*p* = 0.029), financial challenges (*p* = 0.019), infant health challenges (*p* = 0.022), high/moderate stress scores (*p* < 0.001), and being languishing or moderately mentally healthy (*p* = 0.007) were also associated with severe/moderate perinatal anxiety.

### Qualitative analysis

#### Worries, challenges, and lockdown benefits

Qualitative responses to open-ended questions showed that women’s most cited worries were related to COVID-19 health and safety across all surveys (mean: 24.4%). Participants noted concerns about when lockdowns would end, when they would see their family again, border closures, and lack of social contact (Table 4).

**Table 4:** Worries, Challenges and Benefits of Lockdown qualitative responses

Variables	Mean, Count (%)	Survey 1	Survey 2	Survey 3	Survey 4	Survey 5	Survey 6
*Worries (n* = *226)*							
COVID-19 health and safety	483 (24.4)	173(25.2)	120(28.1)	83(27)	47(19.3)	32(19.8)	28(18.7)
General family /parent health	285 (14.4)	103(15)	61(14.3)	33(11)	26(10.7)	16(10)	24(10)
Financial	234 (11.8)	90(13.2)	51(11.9)	36(12)	37(15.2)	27(16.7)	15(10)
Parenting and relationships	314 (15.9)	100(14.6)	70(16.3)	52(17)	43(17.6)	25(15.4)	24(16)
Infant/child health	215 (10.9)	70(10.2)	36(8.4)	33(11)	40(16.4)	21(13)	15(10)
Day to day household/living	230 (11.6)	59(8.6)	47(11)	37(12)	34(13.9)	24(14.8)	29(19.3)
Returning to work	136 (6.9)	47(6.9)	28(6.5)	24(8)	11(4.5)	12(7.4)	14(9.3)
Breastfeeding	52 (2.6)	33(4.8)	11(2.6)	6(2)	2(0.8)	0(0)	0(0)
Lack of support	22 (1.1)	9(1.3)	2(1.9)	6(2)	2(0.8)	2(1.2)	1(0.07)
Other	7(0.35)	0(0)	1(0.2)	1(0.33)	2(0.8)	3(1.9)	0(0)
Total	1978	684	427	311	244	162	150
*Challenges (n* = *224)*							
COVID-19 health and safety	94 (11.4)	38 (13)	28(16)	15(12.5)	6(6.1)	3(4.3)	4(5.9)
General family /parent health	115 (14.0)	36(12.5)	26(14.5)	16(13.3)	16(16.3)	12(17.4)	9(13.4)
Financial	37 (11.9)	12(4.2)	7(3.9)	7(5.8)	6(6.1)	2(2.9)	3(4.5)
Parenting and relationships	218 (26.5)	88(30.4)	40(22)	28(23.3)	27(27.6)	18(26.1)	17(25.4)
Infant/child health	112 (13.6)	38(13.2)	24(13.4)	14(11.7)	17(17.3)	9(13)	10(14.9)
Day to day household/living	136 (16.5)	27(9.3)	32(17.8)	29(24.2)	18(18.4)	15(21.7)	15(22.4)
Returning to work	28 (3.4)	6(2.1)	7(3.9)	4(3.3)	2(2)	4(5.8)	5(7.5)
Breastfeeding	59 (7.2)	35(12.1)	12(6.7)	3(2.5)	4(4.1)	2(2.9)	3(4.5)
Lack of support	20(2.4)	9(3.1)	3(1.7)	4(3.3)	1(1)	2(2.9)	1(1.5)
Other	3	0(0)	0(0)	0(0)	1(1)	2(2.9)	0(0)
Total	822	289	179	120	98	69	67
*Lockdown benefits (n* = *214)*							
Reduced stress/pressure	277 (36.3)	109(40)	58(36)	35(31.1)	37(38.9)	21(31.4)	17(32)
Family time	160 (20.9)	66(24)	36(22)	25(22.2)	11(11.6)	12(17.9)	10(19)
Working from home	49 (6.4)	20(7)	9(5.6)	7(6.3)	5(5.3)	5(7.5)	3(5.6)
Partner support	51 (6.7)	18(7)	7(4.3)	9(8)	7(7.4)	6(9)	4(7.5)
Not in lockdown	52 (3.5)	3(1)	14(8.6)	11(9.8)	10(10.5)	5(7.5)	9(17)
No change/worse	43 (5.6)	13(5)	9(5.6)	4(3.6)	10(10.5)	5(7.5)	2(3.7)
Health	29 (3.8)	10(4)	7(4.3)	4(3.6)	3(3.2)	2(3)	3(5.7)
Online services	27 (3.5)	7(3)	5(3.1)	6(5.4)	6(6.3)	2(3)	1(1.9)
Safety	21 (2.7)	11(4)	5(3.1)	1(0.9)	0(0)	3(4.5)	1(1.9)
Breastfeeding	9 (1.2)	3(1)	2(1.2)	1(1)	1(1)	1(1.5)	1(1.9)
Other	45 (5.9)	14(5)	10(6.2)	9(8)	5(5.3)	5(7.5)	2(3.7)
Total	763	274	162	112	95	67	53

“Challenging being in lockdown and not being able to share our newborn with our family and friends.”

“That I won’t be able to see extended family this year and they won’t get to know our daughter.”

The second most cited worry (15.9%) related to parenting and relationships, including their relationship with their husband or partner and whether they were good parents.

“Am I doing enough for my baby.”

General family health was also cited (14.4%), where women noted their mental health and sleep as a concern and the health of their extended family.

“Being unwell at the moment but still having to manage a household and look after children.”

Participants reported challenges experienced in the previous 2 weeks most frequently with parenting and relationship difficulties (26.5%), including sleep, changing relationships, and difficulty parenting without support.

“Birthing new bub without my husband present as he couldn’t get home due to the Queensland border closures despite being in a Covid free part of northern New South Wales.”

“Only sleep. Youngest either up every hour to get resettled (fed back to sleep) or just decides he’s awake and wants to play. Ultimately both scenarios wake the toddler, and then we’re all awake.”

Mothers frequently cited two closely related benefits of the lockdown: reduced stress/pressure (36.3%) and increased family time (20.9%). Women reported less pressure to deal with visitors and more time to slow down with the family, bond with their newborn, and gain extra support from their partners, who often worked from home. On the other hand, extended lockdowns were no longer beneficial as they reduced immediate family and other support networks.

“Pandemic aside, lockdown has been very enjoyable for our family - so much time together with our new baby, which we never expected. Cohesive family relationships and time to take stock.”

“It takes a village to raise a child and our entire village has been removed from us.”

## Discussion

During the COVID-19 pandemic, breastfeeding mothers in Australia and NZ maintained breastfeeding rates similar to pre-pandemic levels. Nonetheless, women experienced common challenges, including low milk supply, which contributed to the earlier cessation of full breastfeeding and mental health challenges that persisted over time. Perceived poor infant sleep was a significant factor associated with stress, perinatal anxiety, mental wellbeing, and breastfeeding status. Although mothers initially reported that lockdowns helped with family bonding, prolonged lockdowns adversely affected social and family support. The results highlight the changing dynamic of the pandemic for breastfeeding women and indicate that access to adapted perinatal care, with face-to-face and telehealth services for lactation and mental health, remains critical for maternal wellbeing.

Our study population demonstrated high full breastfeeding rates up to 4 months postpartum (87%), which decreased to 37% at 6 months, similar to pre-pandemic Australian breastfeeding rates (6 months: 29%) [33], suggesting COVID-19 did not majorly change breastfeeding. Nonetheless, we found several factors were related to a shorter breastfeeding duration. Perceived low milk supply was associated with a shorter duration of breastfeeding, consistent with our cross-sectional study [19] and previous literature [34–36]. This finding highlights the need for professional lactation support for women with perceived milk supply issues across lactation. While breastfeeding support and low milk supply issues are relevant during the establishment of lactation, they remain important across the first 6 months as mothers may perceive infant developmental changes or unsettled periods as indicative of low milk supply. Similarly, women may lack confidence in breastfeeding despite signs of sufficient milk intake [37, 38] such as adequate infant growth, urine and stool output, and calm periods after breastfeeding [38]. Since improving mothers’ breastfeeding confidence is associated with longer, more positive breastfeeding experiences, ongoing education is required to enhance mothers’ breastfeeding self-efficacy with access to clinical care when there are concerns about milk supply [38, 39].

Shorter full breastfeeding duration was also associated with longer daytime sleep, overtime (in later surveys), and more days pregnant during the pandemic. Shorter breastfeeding duration with longer daytime sleep and overtime was likely related to infant age. Older infants typically demonstrate more extended day nap durations [40], potentially causing less frequent feeding during the day and increased night-time wakefulness. Similarly, as infants were older in later surveys, mothers were more likely to introduce other foods, which likely explain the cessation of full breastfeeding. We have previously found that more days pregnant during the pandemic is associated with poorer maternal mental health [19], suggesting an interplay between perinatal mental health and breastfeeding duration during COVID-19. Indeed, studies in Italy and the USA have shown decreased exclusive breastfeeding rates during lockdown periods when access to services and maternity care is limited [9, 41]. Pandemic restrictions affecting maternal mental health [11, 14–16] and family dynamics in the home [42] may have also influenced breastfeeding choices in our study. Despite these findings, the study population included highly educated women who often worked in healthcare, which may have inadvertently contributed to the high breastfeeding rates and potential knowledge surrounding the pandemic and the benefits of continued breastfeeding.

Mothers experienced adverse mental health outcomes during the pandemic, which persisted over time. Around 40% of mothers consistently displayed medium stress levels, with more than 50% showing mild/moderate perinatal anxiety and moderate mental health. Our qualitative data reiterated these findings, with mothers citing significant worry about COVID-19, likely exacerbating parenting concerns and maternal stress. While initial lockdowns reduced pressure and enhanced family bonding, prolonged lockdowns lost their benefits for some, forcing ongoing separation from immediate and extended families and support networks. These data closely match our cross-sectional study [19] and demonstrate that the COVID-19 pandemic has exacerbated challenging circumstances and stress when navigating parenting, family life, and relationships.

Our previous cross-sectional study and others have highlighted similar maternal challenges and stress in response to the pandemic [11, 43, 44]. While many mothers have experienced multiple new stressors during the pandemic, some may be at higher risk of poor mental health. Others may have support or protective factors such as resilience to mitigate such stressful changes [45, 46]. In China, Italy, and the Netherlands, pandemic-related work and life stress, family conflict, and resilience contributed to maternal mental health during COVID-19 [11]. Although young single and unemployed mothers with poor health are at greater risk of mental health issues, highly educated mothers with high family incomes were also vulnerable in the Netherlands and China. This may be explained by these mothers experiencing disruptions to their usual support systems, including daycare, house cleaning, and other paid services, and may also be relevant to our highly educated cohort [11]. Together, these results emphasise the importance of individualising clinical and mental health care during the pandemic by considering mothers’ circumstances, including physical health, socioeconomic status, and culture [11, 46].

The majority of the mothers in our study perceived their infant’s sleep as a problem; this was associated with higher stress. Those who did not consider infant sleep as problematic reported longer durations of infant night sleep and full breastfeeding, and lower levels of mental health issues, stress, and perinatal anxiety. Our findings corroborate another Australian-based study, where 46% reported infant sleep as a problem that also predicted maternal depression [47]. Associations between reported problematic infant sleep and poorer maternal health are complex. While poor maternal sleep quality may exacerbate postpartum anxiety and depression [48], women with poor mental health are more likely to perceive their infant’s sleep as problematic [49]. Disrupted sleep, while typical during early parenting, can impact a mother’s enjoyment of her baby, functioning, and mental health [49]. Attitudes and beliefs about infant sleep inform parental expectations, resulting in perceived sleep problems when the infant’s sleep pattern does not match expectations [50]. Traditional behavioural infant sleep interventions that include delayed responses to cues and feed-sleep routines do not improve infant or maternal outcomes and may result in unintended consequences [51]. Anticipatory antenatal and early postnatal education that includes typical infant sleep patterns may assist parents in forming realistic expectations. Approaches that promote parents’ understanding of normal infant sleep patterns while supporting their own sleep and wellbeing are reported to be easy to implement and helpful [52, 53]. As mental health challenges and concerns about infant sleep are amplified during times of increased stress [54], such approaches must be made widely available through a variety of media, including telehealth, to support women regardless of the availability of face-to-face professional support.

Our study was limited for several reasons. Our sample included breastfeeding women who reported high rates of full breastfeeding, suggesting that our population was highly motivated to breastfeed, which may not reflect all breastfeeding women in Australia and NZ. Women also started the initial survey at differing time points and infant ages and differed in the number of surveys they completed. Finally, we were unable to determine if women ever introduced formula in the early days, and thus we could only capture full breastfeeding rather than exclusive breastfeeding rates.

## Conclusion

Breastfeeding mothers in Australia and New Zealand have experienced new stressors and challenges affecting their mental wellbeing during the COVID-19 pandemic. Over 6 months, mothers continued to breastfeed while facing mental health and sleep challenges potentially intensified by the pandemic. During lockdowns, the initial benefits of family time seemed to be overshadowed by the negative impact of limited or absent extended family support. The mothers’ individual situation is important when considering lactation, mental health, and social care. Adaptable perinatal care, including telehealth and in-person support, and allowing new mothers access to their social support networks is critical to enabling continued breastfeeding and the mental wellbeing of mothers during the pandemic.

## Supplementary Information

Below is the link to the electronic supplementary material.

Supplementary file1 (XLSX 38 KB)

## Abbreviations

FAD: McMaster Family Assessment Device
GF6 +: General Functioning subscale
MHC-SF: Mental Health Continuum-Short Form
NZ: New Zealand
PASS: Perinatal Anxiety Screening Score
PSS: Perceived Stress Score
